# Does patient or clinician gender modify the efficacy of a primary care brief intervention for adolescent alcohol use?

**DOI:** 10.1186/1940-0640-10-S2-O16

**Published:** 2015-09-24

**Authors:** Lilia D'Souza-Li, John R Knight, Lon Sherritt, Jesse Boggis, Sion K Harris

**Affiliations:** 1 Boston Children's Hospital Center for Adolescent Substance Abuse Research, Boston, MA, 02115, USA; 2 Department of Pediatrics, Harvard Medical School, Boston MA, 02115, USA; 3 Department of Pediatrics, Faculty of Medical Science, University of Campinas, Campinas, SP, 13087-970, Brazil

## Background

A previous large, multi-site trial showed that computer-facilitated screening and physician brief advice (cSBA) was an effective way to reduce adolescents' alcohol use[1]. What is unknown is whether the intervention effect varies by patients' or physicians' characteristics, such as gender. We assessed whether patient and physician gender moderates cSBA effectiveness.

## Material and methods

We analyzed a subset of data from a quasi-experimental, asynchronous effectiveness trial of 12-18 y/o patients at primary care sites. An initial 18-month Treatment as Usual (TAU) phase was followed by a 1-hour physician training and an 18-month cSBA phase with computerized screening, immediate feedback and information on the health risks of drugs, follow by physicians brief advice. Adolescents rated their visit and physician immediately post-visit. Only data for physicians with >5 patients in each study arm were included. We conducted stratified multiple logistic regression modeling with adjustment for known covariates and within-site clustering. Endpoints were past 3- and 12-month alcohol at follow-ups.

## Results

Subjects: 20 physicians (11 females; 85% pediatricians) and 1158 patients, mean age 15.6+2.0 yrs. Youth-provider connectedness was high (median score 32 [IQR 29-34] out of 35 max). However, female physicians' patients scored significantly higher on youth-provider connectedness than patients of male physicians (p<0.0001), regardless of patient gender. The 3-month effect of cSBA on adolescent alcohol use was stronger among girls and female physicians (Table 1).

**Table 1:** Adjusted relative risk ratios (ARRR) comparing cSBA vs. TAU adolescent alcohol use rates at 3 and 12 months post-visit.

	3 MONTHS		12 MONTHS	
	ARRR	95%CI	ARRR	95% CI
Girl patient (n=546)	0.47*	0.25-0.87	0.82	0.52-1.31
Boy patients (n=612)	0.54	0.26-1.19	0.71	0.45-1.11
Female doctors(n=658^1^)	0.41*	0.22-0.76	0.63	0.38-1.03
Male doctors (n=500^1^)	0.62	0.30-1.29	0.77	0.50-1.17

*p<0.05; ^1^ Number of patients

## Conclusion

Physician advice regarding alcohol use may be particularly effective for girls within the context of an ongoing relationship with their physician, and when delivered by female physicians whose care is associated with higher patient-provider connectedness.
